# Strengthening primary health care in low- and middle-income countries: furthering structural changes in the post-pandemic era

**DOI:** 10.3389/fpubh.2023.1270510

**Published:** 2024-02-14

**Authors:** J. C. Alegre, Suneeta Sharma, Farley Cleghorn, Carlos Avila

**Affiliations:** The Palladium Group, Washington, DC, United States

**Keywords:** primary health care, universal health coverage, gamechangers, local health systems, health outcomes

## Abstract

Strengthening primary health care (PHC) is the most cost-effective approach in low- and middle-income countries (LMICs) to achieve sustainable universal health coverage (UHC), protect against health shocks, and promote health and wellbeing for all people. It has been 45 years since PHC was put on the global agenda followed by multiple efforts to advocate for more funding and improved performance of PHC. Yet, investment in PHC is still insufficient and overall performance of PHC systems is weak in LMICs, resulting in increased vulnerability and poor health outcomes especially among marginalized populations. As countries recover from the COVID-19 pandemic, which exposed the fragility of PHC platforms, it is imperative to go beyond advocacy for PHC investments and make systemic changes to strengthen PHC as the foundation of resilient and equitable health systems. We propose five *gamechangers* to facilitate structural changes for strengthening PHC through a focused health systems approach: (i) integration of client-centered health services at PHC level; (ii) digitization of PHC services; (iii) efficiency gains invested in essential health services; (iv) strengthening management practices for PHC at district and facility levels; and (v) advancing community engagement for PHC. To be successful, the implementation of the *gamechangers* must be contextualized and focus on achieving sustainable health outcomes, and therefore use implementation approaches that link essential health services to health outcomes. Through this way countries will maximize the possibility of achieving UHC and attaining the ambitious health targets of the Sustainable Development Goals (SDGs) by 2030.

## Introduction

The fundamental premise of primary health care (PHC) is that all people, everywhere, have the right to achieve the highest attainable level of health. The World Health Organization (WHO) defines PHC as a whole-of-society approach to effectively organize and strengthen national health systems to bring services for health and wellbeing closer to communities by using three components: integrated health services to meet people’s health needs throughout their lives; addressing the broader determinants of health through multisectoral policy and action; and, empowering individuals, families and communities to take charge of their own health (1).

**Figure 1:**
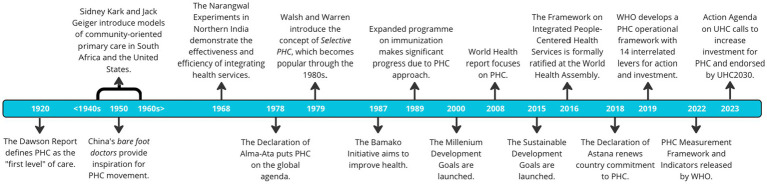
Evolution of primary health care in the last century.

PHC has been on the radar screen of multiple stakeholders for more than a century since the Dawson Report defined PHC as the “first level” of care in 1920 in the U.K. – see Figure 1 (2–4). Several countries tested models of PHC and community outreach, perhaps more notably in China, India, and Latin America (5–7). Years later at the Alma Ata Declaration in 1978, country governments, donors, implementing partners, policy makers and researchers advocated for strengthening PHC (8, 9). Recent estimates of PHC spending range between $15 and $60 *per capita* in LMICs (10). In contrast, PHC interventions in LMICs are projected to cost $97 *per capita* to achieve 80% coverage (11). PHC is a key component in all high-performing health systems and constitutes a foundation for universal health coverage (UHC) (12). While there has been significant progress in achieving positive health outcomes and UHC, PHC platforms remain fragile and catastrophic out-of-pocket health spending continues to be a major barrier to access healthcare services (13). The global incidence of catastrophic spending at the 10% threshold has been estimated at 11.7%, which is equivalent to 808 million of individuals and families paying for their health are reduced to a state of financial ruin: impoverished and bankrupted globally (14). The incidence of catastrophic health expenditure correlates negatively with the share of total government health spending. Higher proportions of health expenditure channelled through social security and other government financial protection arrangements are protective against both catastrophic and impoverishing health expenditure (15). Currently 54 countries are still off-track to achieving health-related SDG targets by 2030 (16) and more will most likely fall behind due to the adverse effects of COVID-19 in health, education, and overall economic growth.

While many funders and governments have invested and delivered successful and promising approaches for strengthening PHC, multiple challenges remain and hinder the full realization of PHC platforms. The effects of the global COVID-19 pandemic have further exacerbated the gaps in PHC. The pandemic has revealed how precarious PHC platforms are for the provision of essential health services, let alone the accomplishment of UHC after many attempts over the last century (Figure 1). This situation has re-surfaced the importance of strengthening health system functions, with a particular attention to strengthen PHC (17), to create sustainable, equitable, and resilient health systems able to provide health for all and absorb future shocks and long-standing health system stressors.

### Need of a new implementation approach

A more actionable approach for strengthening PHC in LMICs is urgently needed given the negative effects we expect from new shocks and health system stressors that LMICs will most likely face in the short, medium, and long term (18). We propose five *gamechangers* to bring targeted structural changes, creating the conditions for a more sustainable, effective functioning of PHC platforms, to improve health outcomes and save lives.

### Gamechanger 1: integration of client-centered services at PHC level

Governments can no longer afford missing the opportunities that PHC platforms offer for integrated health services, better quality and patient experience, fewer hospital and emergency room visits, and overall, a value-based cost of care. Prevention, early detection, and treatment of a disease in PHC is more cost-effective than the treatment of a disease in a hospital setting. And donors of global health programs should review their funding models and reconfigure stand-alone funding streams that hinder holistic approaches to address health needs, particularly of marginalized populations and vulnerable groups. While integration is not a novel concept, we propose prioritizing three integrated approaches amid limited financial resources and low capacity of health systems that many LMICs face. First, adopting a laser focus approach on the integration of health service delivery for communicable and non-communicable diseases addressing the client’s health needs holistically. This includes the necessary health system functions integration necessary for those services to be effectively delivered. Second, as the world moves on with the post-pandemic phase, reprioritize the integration of routine immunization services with ongoing (i.e., COVID-19) and upcoming vaccines; this will not only alleviate shrinking resources for COVID-19 vaccination activities but will also revert decreasing immunization rates registered globally. And third, integration of outreach and community-based activities into facility-based PHC. This is a key link that leverages the role of community actors, including community health workers (CHWs) to bring trust to public health systems.

### Gamechanger 2: digitization of PHC data and services

Digitization unlocks increased access to health data, the use of which can inform more precisely targeted and adaptive decision-making at all levels of health care (19). Digitization of PHC services should provide a great opportunity to improve the supply and demand of essential health services in low-resource settings. We propose three specific approaches that LMICs can benefit, leveraging a plethora of pilots (20–22) conducted over the past few decades. First, expand the availability of electronic records for PHC services at health facilities, including health centres that have the basic digital infrastructure for doing so; penetration of electronic health records remain significantly low in LMICs (23); the proposed approach will maximize access to health data by clients and providers for improving the provision of essential health services. Second, epidemiological profiling of populations within a catchment area through digital tools (i.e., establish electronic census of pregnant women and CHWs to improve surveillance of maternal and neonatal health, particularly to support addressing complications during pregnancy, delivery and postpartum and begin addressing the growing demand for NCD prevention); we believe that leveraging the trust of CHWs in their communities and digital health solutions for community surveillance should address the enduring barriers of access, availability and demand for essential health services in low-resource settings. And third, telemedicine, especially teleconsultation and the use of digital tools where appropriate to triage clients.

### Gamechanger 3: increased efficiency at the PHC level

PHC platforms have been facing significant gaps in health financing programs for multiple reasons, including the perceived notion of the inefficient use of existing resources. A multi-country study reported a positive correlation between the share of health expenditure in the public budget and the performance of the health system (24). And another report comparing 36 African countries found that only 58% of healthcare systems were efficient; however, evidence of PHC efficiency in developing countries is lacking. A study of determinants of efficiency of PHC in China suggests that while increasing financial support for PHC is a priority, it needs to be complemented by a reasonable reimbursement design, appropriate payment methods, and comprehensive social health insurance policies (25). The digitization and wide data availability will facilitate a shift toward PHC outcomes-based funding payments. Population-based provider payment mechanisms, such as capitation, should be the cornerstone of financing for people-centred PHC (26). New funding models will be linked to meeting specific targets and measures related to achieving quality and sustainable health outcomes. While we do not neglect the need to advocate for the assignation of more funds for PHC, we propose focusing on improving the use of existing resources for PHC. These include: [i] expanding strategic purchasing for PHC services; [ii] enhancing budget execution for PHC programs, especially at district level; and [iii] improving the allocation of resources for PHC services with a renewed effort towards promotion and preventive health services. There is compelling evidence that when financial incentives are linked to quality improvement and community involvement is strong then PHC performance is improved (27). Increasing efficiency should also facilitate the inclusion of the private sector with more sustainable public/private partnerships for PHC.

### Gamechanger 4: strengthening management practices for PHC at the district and facility levels

Management practices must be strengthened at various levels, including those where management and resource allocation decisions are made. These include strengthening leadership, management, and governance within district health management teams and working upstream with medical and public health management schools to strengthen pre-service training curricula for PHC. Public financing management (PFM) system and rules are crucial for the effective implementation of health financing policy in support of UHC. A strong PFM system ensures higher and more predictable budget allocations, reduces fragmentation in revenue streams and funding flows, supports timely budget execution, and better financial accountability and transparency (28). PHC managers need to be empowered with information, capacity, and autonomy, and held accountable for results and actions. In addition, data demand and use for PHC should be adopted at all levels, focusing on data-informed management decisions at district and facility levels. Also, there is strong need to improve oversight and accountability of PHC initiatives at national level, regardless the level of decentralization of health systems. Lastly, the human resource aspect for PHC is crucial. We propose focusing on full recognition of CHWs within the health system and adopting sustainable remuneration strategies for them.

### Gamechanger 5: advancing community engagement for PHC

This is perhaps the most important yet neglected aspect to support a strong and functioning PHC system. LMICs should make deliberate efforts to fully engage communities in the design and implementation of client-centered integrated PHC services. CHWs who are trusted members of their communities are best suited for establishing stronger links between the supply of and demand for PHC services. On the supply side, CHWs can play a key role in revamping outreach programs of health facilities for promoting community-based approaches like integrated community case management (iCCM). On the demand side, CHWs can further support increasing awareness, knowledge, and agency among marginalized populations to increase adoption of promotion and prevention practices for good health. This approach will facilitate fit-for-purpose and sustainable solutions—including policy and service provision changes—for delivering essential health services and meet the health needs of populations, especially of marginalized and vulnerable groups. Engaging communities and responding to their health needs based on their feedback will maximize prioritization and sustainability.

## Discussion

The implementation of the proposed five *gamechangers* requires a health systems approach. However, there is no “one-size-fits-all” implementation model for strengthening health system functions to support PHC platforms. The proposed *gamechangers*, while not entirely novel, must be contextualized to local priorities, needs, and resources to address most of the structural challenges that hinder the provision of quality PHC services in low-resource settings. The post-pandemic era offers a new opportunity to revitalize PHC as COVID-19 has revealed the fragility of PHC platforms. For these approaches to be successful, they must be feasible, sustainable, and linked to positive health outcomes. This is especially important for marginalized and vulnerable populations where health needs and gaps in the provision of PHC services are the greatest in LMICs. The postpandemic era presents a golden opportunity to be pragmatic in bringing sustainable solutions that if delivered properly will drive equitable and high-performing PHC platforms. Lastly, a common framework for monitoring and evaluating the implementation of the *gamechangers* will require special attention, including comparable data, transparency of measurements, and accountability to local stakeholders. The implementation of the *gamechangers* may bring the health-related SDG targets within reach by 2030.

## Author contributions

JCA: Conceptualization, Visualization, Writing – original draft, Writing – review & editing. SS: Writing – review & editing. FC: Writing – review & editing. CA: Supervision, Writing – review & editing.
